# Vitamin C and Heart Health: A Review Based on Findings from Epidemiologic Studies

**DOI:** 10.3390/ijms17081328

**Published:** 2016-08-12

**Authors:** Melissa A. Moser, Ock K. Chun

**Affiliations:** Department of Nutritional Sciences, University of Connecticut, Storrs, CT 06269, USA; melissa.a.moser@uconn.edu

**Keywords:** vitamin C, cardiovascular disease, observational cohort studies, clinical trials, meta-analyses

## Abstract

Vitamin C is a powerful dietary antioxidant that has received considerable attention in the literature related to its possible role in heart health. Although classical vitamin C deficiency, marked by scurvy, is rare in most parts of the world, some research has shown variable heart disease risks depending on plasma vitamin C concentration, even within the normal range. Furthermore, other studies have suggested possible heart-related benefits to vitamin C taken in doses beyond the minimal amounts required to prevent classically defined deficiency. The objective of this review is to systematically review the findings of existing epidemiologic research on vitamin C and its potential role in cardiovascular disease (CVD). It is well established that vitamin C inhibits oxidation of LDL-protein, thereby reducing atherosclerosis, but the cardiovascular outcomes related to this action and other actions of vitamin C are not fully understood. Randomized controlled trials as well as observational cohort studies have investigated this topic with varying results. Vitamin C has been linked in some work to improvements in lipid profiles, arterial stiffness, and endothelial function. However, other studies have failed to confirm these results, and observational cohort studies are varied in their findings on the vitamin’s effect on CVD risk and mortality. Overall, current research suggests that vitamin C deficiency is associated with a higher risk of mortality from CVD and that vitamin C may slightly improve endothelial function and lipid profiles in some groups, especially those with low plasma vitamin C levels. However, the current literature provides little support for the widespread use of vitamin C supplementation to reduce CVD risk or mortality.

## 1. Introduction

In their 2004 Science Advisory Report, the American Heart Association stated that the current literature does not support the use of antioxidant vitamin supplements for the prevention or treatment of cardiovascular disease (CVD) [1]. Although the research conducted in this area is not uniformly positive, a substantial body of work has hinted at a possible reduction in CVD risk associated with antioxidant intake. Of particular interest to this review is the potential role of vitamin C in heart health. Vitamin C’s functions as an antioxidant and as an enzyme cofactor are well established, but the ways in which these functions may modify chronic disease risk are less well established.

The belief that vitamin C may benefit heart health has stemmed from multiple pieces of evidence and lines of reasoning. First, much work has documented the beneficial effects of fruit and vegetable consumption on heart health, which has led to the hypothesis that, among other nutrients, vitamin C may be partially responsible for this relationship. Importantly, the link between fruit and vegetable consumption and improved cardiovascular outcomes has primarily been established through cohort studies, as randomized controlled trials in this area are scarce. Nevertheless, the wealth of evidence accumulated from these epidemiological and cohort studies has consistently demonstrated the cardiovascular benefits of fruits and vegetables. For example, in the Nurse’s Health Study (NHS) and the Health Professionals Follow-Up Study (HPFS), which together looked at over 126,000 healthy adults, it was shown that individuals in the highest quintile of fruit and vegetable intake had a relative risk for coronary heart disease (CHD) of 0.8 (95% confidence interval [CI]: 0.69–0.93) compared to those in the lowest quintile of intake [2]. Of note, green leafy vegetables and vitamin C-rich fruits contributed most to the apparent protective effect of total fruit and vegetable intake. Another cohort of 1725 men in Sweden showed similar benefits of fruits and vegetables [3]. Further, a meta-analysis of 13 independent cohorts including 278,459 total participants showed that compared with individuals who had less than three servings per day of fruits and vegetables, the pooled relative risk of CHD was 0.93 (95% CI: 0.86–1.00) for those consuming three to five servings per day and 0.83 (95% CI: 0.77–0.89) for those consuming more than five servings per day. In this study, increased consumption of fruits and vegetables from less than three to more than five servings per day was related to a 17% reduction in CHD risk [4].

The hypothesis that vitamin C may play a role in CVD prevention also draws support from the vitamin’s antioxidant capabilities. The epidemiological evidence relating fruit and vegetable intake to reduced risk of CVD may be explained, at least in part, by antioxidant content, and especially the role of these antioxidants in preventing oxidative changes to LDL [5]. Oxidized LDL is a target for scavenger receptors, which incorporate it into plaque [6]. Therefore, the prevention of LDL oxidation by vitamin C may prevent atherosclerosis, thereby mediating a potential role in CVD risk reduction.

Various other functions of vitamin C may also bolster the hypothesis that vitamin C can reduce cardiovascular risk. For example, vitamin C has been shown to reduce monocyte adhesion to the endothelium [7]. Adhesion of circulating monocytes to endothelial cells is one key in the formation of atheromas, and is considered one of the early signs of the development of atherosclerosis [7]. Additionally, vitamin C has been shown to improve nitric oxide production of the endothelium, which, in turn, increases vasodilation, reducing blood pressure [8]. Furthermore, vitamin C may prevent apoptosis of vascular smooth muscle cells, which helps keep plaques more stable if atherosclerosis has developed [9].

The requirement for dietary vitamin C is based on its role as an antioxidant, and was determined by estimating the quantity of dietary vitamin C needed to maximize its concentration in neutrophils, where it reduces reactive oxygen species produced during phagocytosis [10]. Given that vitamin C may relate to heart disease risk through more than one mechanism, it is possible that the recommended or typical levels of vitamin C intake are incongruous with the intake levels needed to provide protection against CVD. Therefore, even the Recommended Dietary Allowances (RDA) for vitamin C of 75 mg for women and 90 mg for men may be inadequate to obtain potential benefits. Based on a recent analysis of US adults in the National Health and Nutrition Examination Survey (NHANES) data, the mean intake of vitamin C from food alone is above the RDA for both men and women (104.6 ± 3.4 mg and 86.6 ± 2.7 mg, respectively) [11]. Many of the clinical trials testing hypotheses related to vitamin C and CVD have used supplemental vitamin C in doses of 500–1000 mg per day. Therefore, if vitamin C does alter cardiovascular risk, questions still remain regarding the relevant mechanisms, the differential effects of supplemental and naturally occurring vitamin C, and the recommended intakes of vitamin C to minimize heart disease risk. This review examines key pieces of literature seeking to answer these questions.

## 2. Methods

This review covers the cardiovascular effects of vitamin C in human studies and attempts to explain the underlying mechanisms involved. A literature search was conducted using three databases: PubMed, Web of Science, and Scopus. Articles were identified in these databases using the search “vitamin C cardiovascular disease”, and additionally using a MeSH search in PubMed: (“Ascorbic Acid” (Mesh)) AND “Cardiovascular Diseases” (Mesh). Other key words added to this search were myocardial infarction, low-density lipoprotein, hypertension, and endothelial function. The reference lists of relevant articles identified in this systematic review were searched manually for additional inclusions. Articles chosen for this review were published by May 2016. Publications identified by these methods were then limited to only those meeting all of the following criteria: (1) randomized controlled trials, observational studies, or meta-analyses examining vitamin C intake (dietary or supplemental) or plasma vitamin C concentrations; (2) studies involving adults aged ≥18 years; (3) studies reporting changes in cardiovascular outcomes or risk factors as an endpoint (mortality, CVD or CHD incidence, lipid profile, endothelial function, blood pressure).

## 3. Observational Cohort Studies

While they are not positioned to establish cause and effect relationships, observational cohort studies have investigated this topic with large groups of individuals and lengthy follow-up periods. The results of these studies, though, have been highly variable (Table 1). Two of the most well -known cohort studies that have investigated the link between vitamin C and CVD are the HPFS and the NHS. Over 121,000 women were followed for 16 years in the NHS. Dietary data were collected from food frequency questionnaires (FFQ) administered at baseline and intervals of every two to four years afterwards [12]. This study found a modest inverse association between vitamin C intake and CHD risk. However, this association was only apparent in women taking supplements containing vitamin C. Importantly, vitamin C intake was not associated with risk of death from CHD. The HPFS, an all-male study designed to complement the NHS, showed even less promise for vitamin C. In their analysis of 39,910 men, researchers found an inverse association between intake of the antioxidant vitamin E and the risk of CHD, but could not demonstrate the same for vitamin C intake. Additional analysis for supplemental vitamin C also showed no significant association with total CVD mortality after adjustment for other CVD risk factors [13].

While these two cohorts examined vitamin C intake in healthy adults, others have examined the relationship between diet and heart disease risk in other populations, including diabetics. In an analysis that combined 520 diabetic participants from the Insulin Resistance Atherosclerosis Study and 422 diabetics from the San Luis Valley Diabetes Study, vitamin C was not associated with CVD risk factor status [18]. The Iowa Women’s Health Study Cohort, which included 1923 diabetic postmenopausal women, found that higher intakes of total vitamin C from supplements and food were associated with an increased relative risk of coronary artery disease and with death from CVD. When dietary and supplemental vitamin C were analyzed separately, only supplemental vitamin C showed a positive association with mortality from CVD [19].

Although these major cohorts largely failed to show a protective effect of vitamin C, or demonstrated possible harm in certain populations, multiple cohort studies have shown that higher plasma vitamin C concentrations may be related to reduced heart disease risk. The European Prospective Investigation into Cancer and Nutrition (EPIC) Study prospectively examined a cohort of 19,496 men and women who were separated into gender-specific quintiles of plasma vitamin C. After a four-year follow-up it was determined that plasma ascorbic acid was inversely associated with the risk of heart failure, as well as all-cause mortality and mortality from CVD and ischemic heart disease [14,15]. Interestingly, the majority of participants in the lowest quintile of vitamin C intake were above the threshold for vitamin C deficiency, demonstrating that this inverse relationship may hold true even within the range of clinically normal vitamin C concentrations. An analysis of 1605 men from the Kuopio Ischaemic Heart Disease Risk Factor Study reached similar conclusions. Of these participants, 91 (5.7%) were found to be deficient in vitamin C (plasma concentrations of less than 11.4 μmol/L). After adjusting for age, year examined, and season of the year examined, men who were deficient in vitamin C had a relative risk of acute myocardial infarction of 3.5 (95% CI: 1.8–6.7) compared to those who were not deficient [17]. It should also be considered, though, that the men deficient in vitamin C were more likely to smoke, consume alcohol, be of a lower socioeconomic group, have lower dietary iron, and have higher systolic blood pressure than those without the vitamin deficiency.

The cardiovascular benefit of adequate intakes and plasma concentrations of vitamin C was also demonstrated in an analysis of 2884 participants in the Coronary Artery Risk Development in Young Adults Study (CARDIA) [16]. The hazard ratio for the development of hypertension per 19.6 μmol/L (1 standard deviation) higher plasma vitamin C was 0.85 (95% CI: 0.79–0.92). Additionally, it was found that dietary vitamin C intake, but not intake from supplements, was inversely related to the development of hypertension.

Overall, these major cohort studies have produced variable results. While several have shown no relationship between vitamin C and CVD risk, one supports the use of vitamin C supplements in healthy adults, and another suggests increased risk to diabetic patients when using vitamin C supplements. It is fairly well established, though, that low plasma concentrations of vitamin C are predictive of heightened CVD risk.

## 4. Clinical Trials

Major clinical trials have largely been unable to demonstrate a benefit of vitamin C in heart health, either in healthy populations or in those with existing heart disease or related risk factors (Table 2). The Physicians Health Study II was one key study that showed no cardiovascular benefit of vitamin C to healthy men. In this randomized, double-blind, placebo-controlled, 2 × 2 × 2 × 2 factorial trial, the effects of vitamin E, vitamin C, and a multivitamin on the prevention of CVD were evaluated in 14,641 males. During a mean follow-up of eight years, it was determined that long-term daily supplementation of 500 mg of vitamin C did not reduce the primary endpoint of incidence of major cardiovascular events. Vitamin C did not reduce total myocardial infarction, total stroke, cardiovascular death, congestive heart failure, total mortality, angina, or coronary revascularization [20].

Another primary prevention trial conducted in healthy adults was the Supplementation en Vitamines et Minéraux Antioxydants (SU.VI.MAX) study. In this trial, 8112 healthy men and women received either a placebo or an antioxidant vitamin blend containing 120 mg vitamin C, 30 mg vitamin E, 6 mg beta-carotene, 100 μg selenium, and 20 mg zinc. As was shown with vitamin C alone in the Physicians Health Study, participants randomized to the antioxidant blend used in this study experienced no change in ischemic CVD incidence compared to the placebo [21].

To investigate the effects of vitamin C in females with a history of CVD and in those with multiple cardiovascular risk factors, the Women’s Antioxidant Cardiovascular Study supplemented 8171 women with vitamin C, vitamin E, beta-carotene, or a placebo. With a mean follow-up of 9.4 years, it was shown that there was no effect of 500 mg daily ascorbic acid supplementation on cardiovascular events [22]. Similarly, the Women’s Angiographic Vitamin and Estrogen (WAVE) trial was a study of 423 postmenopausal women with at least one 15%–75% coronary stenosis at baseline. Participants receiving 400 IU of vitamin E twice daily plus 500 mg of vitamin C twice daily had a 0.044 mm/year worsening in coronary progression. Those taking a placebo experienced milder coronary progression of 0.028 mm/year. Death, nonfatal myocardial infarction, or stroke occurred in 26 participants taking the antioxidant vitamins compared to 18 taking the placebo [23].

A small study conducted last year on 64 Palestinian men and women who were obese and hypertensive and/or diabetic yielded similar results [24]. Participants were randomized into an experimental group, which received 500 mg vitamin C twice daily, or a control group that received no supplement. After eight weeks, those taking daily vitamin C supplements experienced no significant improvements in total cholesterol or triglyceride levels compared to the control.

Another trial investigating the effects of antioxidants on CVD risk was the HDL-Atherosclerosis Treatment Study (HATS). In this three-year double-blind trial, 160 participants with CHD, low HDL cholesterol, and normal LDL cholesterol were assigned to one of four groups: Simvastatin plus niacin, antioxidants (800 IU vitamin E, 1000 mg vitamin C, 25 mg beta carotene, and 100 μg selenium), Simvastatin plus niacin and antioxidants, or placebos. The group taking Simvastatin plus niacin experienced a protective increase in HDL, but this was attenuated by concurrent therapy with antioxidants. The antioxidant group experienced no change in mean LDL or HDL, signifying no cardiovascular benefits of the antioxidant blend [25]. Overall, each of these clinical trials showed either no cardiovascular benefit with vitamin C supplementation, or possible harm related to vitamin C supplement use.

## 5. Meta-Analyses

Reviewed above are several major clinical trials that have investigated the effects of vitamin C on cardiovascular health. Other research groups have focused more narrowly on the effects of vitamin C or antioxidant blends on particular markers of cardiovascular health including arterial stiffness, endothelial function, lipid profile, and blood pressure (Table 3).

A 2015 meta-analysis of randomized controlled trials analyzing the effect of antioxidant supplements on arterial stiffness, which included 20 studies and a total of 1909 participants, showed that antioxidant supplements significantly reduced arterial stiffness. However, this benefit was observed only in studies using vitamin E or combined antioxidants, but not with vitamin C alone. Additionally, antioxidant vitamins were shown to be most effective in participants with low baseline plasma concentrations of vitamins C and E [26]. The same research group published a meta-analysis in 2014 of randomized controlled trials analyzing the effect of vitamin C and E supplements on endothelial function. They looked at 46 studies, 17 of which included participants receiving supplements of vitamin C alone. These studies showed a significant improvement in endothelial function, but they had significant heterogeneity, making this result less convincing. Again, the effects were most pronounced in participants with low baseline plasma concentrations of vitamins C and E [27]. A third meta-analysis by this group analyzing the effect of vitamin C on lipid profiles included 40 studies with a total of 1981 participants. Overall, vitamin C was ineffective in improving total cholesterol, LDL, HDL, or triglycerides. Some improvements were seen, however, in certain subgroups. A small reduction in total cholesterol (−0.26 mmol/L) was observed in participants younger than 52 years old and a small increase in HDL cholesterol (0.06 mmol/L) was observed in diabetics [28].

One meta-analysis that showed more promising results with vitamin C supplementation looked at its effects on blood pressure. This analysis looked at 29 trials with a median dose of 500 mg daily vitamin C. The median duration of supplementation in these trials was only eight weeks, but in these short-term studies, it was shown that vitamin C supplementation reduced both systolic and diastolic blood pressure [29]. In addition to the limitation of the short duration of the intervention, this meta-analysis and others are limited by only looking only at supplemental vitamin C.

Overall, these meta-analyses demonstrate that supplementation with vitamin C may improve endothelial function and reduce blood pressure. In concert with the antioxidant vitamin E, but not alone, vitamin C may also reduce arterial stiffness. Although cardiovascular benefits are not consistently seen the in the experimental or observational data, these meta-analyses looking at individual components of heart disease risk lend support to possible mechanisms by which vitamin C may be related to heart disease.

## 6. Conclusions

The current literature provides little support for the use of vitamin C supplementation to reduce heart disease risk. Many cohort studies and randomized trials have shown no relationship between vitamin C intake and heart disease risk, while few have suggested moderate benefits, and some have even suggested slight increases in risk. Importantly, multiple studies have documented increases in cardiovascular risk associated with the use of supplemental vitamin C, even when taken in doses of about 1000 mg per day, which is half of the established Tolerable Upper Intake Level (UL) of 2000 mg [19,23]. Although several studies have shown similar absorption of vitamin C from supplements and from food sources [30,31], the mechanisms behind the apparent differential effects of supplemental and dietary vitamin C require further examination.

Interestingly, meta-analyses examining particular risk factors of heart disease show that vitamin C may favorably affect blood pressure and endothelial function. While these are only single elements of CVD, they do lend support to the hypothesis that vitamin C may play a role in heart disease. Additionally, multiple observational studies have confirmed that CVD risk and mortality are increased in those with the lowest plasma concentrations of vitamin C, even if they are not classified as deficient in this vitamin. All of these studies, however, are limited in several ways, including the use of only supplemental vitamin C, limited follow-up or intervention time, and reliance on self-reported diet or self-reported compliance with interventions.

The lack of consistency within the body of research on this topic has called into question the roles of antioxidants in the human body, and has even cast doubt on the LDL oxidative hypothesis [32,33,34]. A key limitation in understanding the relationship between vitamin C and CVD is the lack of mechanistic studies in humans. Most evidence supporting this link is based on animal studies and observational studies in humans, which both may fail to capture other factors that could play an important mediating role in this relationship. Despite these issues, there is still compelling evidence that warrants continued investigation of the role of vitamin C and other antioxidants in CVD.

## Figures and Tables

**Table 1:** Cohort studies investigating vitamin C and cardiovascular disease (CVD).

Cohort	Population	Mean Follow-up	Size (*n*)	Age (years)	Outcomes
EPIC [14,15]	Healthy men and women	4 years	19,496	45 to 79	Plasma vitamin C inversely related to risk of heart failure and mortality from CVD and ischemic heart disease
CARDIA [16]	Healthy men and women	15 years	2884	18 to 30	Dietary vitamin C inversely related to hypertension
Kuopio Ischaemic Heart Disease Risk Factor Study [17]	Healthy men	5 years	1605	42, 48, 54, or 60	Vitamin C deficiency associated with increased CHD risk
NHS [12]	Healthy women	16 years	85,118	30 to 55	Vitamin C from supplements (but not from foods) associated with lower risk of CHD
HPFS [13]	Healthy males	4 years	39,910	40 to 75	Vitamin C intake not associated with CHD risk
IRAS and SLVDS [18]	Diabetic men and women	4 years	IRAS *n* = 520, SLVDS *n* = 422	IRAS: 40 to 69; SLVDS: 20 to 74	Vitamin C not associated with CVD risk factor status
Iowa Women’s Health Study [19]	Postmenopausal diabetic women	15 years	1923	55 to 69	Supplemental vitamin C intake associated with an increased risk of CVD mortality

EPIC: European Prospective Investigation into Cancer and Nutrition; CARDIA: Coronary Artery Development in Young Adults Study; NHS: Nurses’ Health Study; HPFS: Health Professionals Follow-Up Study; IRAS: Insulin Resistance Atherosclerosis Study; SLVDS: San Luis Valley Diabetes Study.

**Table 2:** Clinical trials investigating vitamin C and CVD.

Clinical Trial	Population	Size (*n*)	Age (years)	Intervention	Trial Duration	Outcome(s)
Women’s Antioxidant Cardiovascular Study [22]	Females with history of CVD or 3 or more CVD risk factors	8171	40 and older	500 mg/day ascorbic acid	9.4 years	No effect on myocardial infarction, stroke, coronary revascularization, or CVD death
Vitamin C Trial in Obese Adults [24]	Obese men and women with hypertension and/or diabetes	64	20–60	500 mg/day ascorbic acid twice daily	8 weeks	No effect on total cholesterol of triglycerides
Physicians Health Study II [20]	Healthy males	14,641	50 and older	500 mg/day ascorbic acid	8 years	No effect on major cardiovascular events, myocardial infarction, stroke, cardiovascular mortality, or total mortality
SU.VI.MAX [21]	Healthy men and women	13,017 (7876 women, 5141 men)	Women 35–60; men 45–60	Daily blend of 120 mg vitamin C, 30 mg vitamin E, 6 mg beta-carotene, 100 μg selenium, and 20 mg zinc	7.5 years	No effect on incidence of ischemic CVD
HATS [25]	Men and women with coronary disease	160	Men under 63; women under 70	800 IU vitamin E, 1000 mg vitamin C, 25 mg natural beta-carotene, 100 μg selenium; and simvastatin and niacin	3 years	No effect on LDL or HDL with antioxidant supplement
WAVE [23]	Postmenopausal women with CVD	423	Postmenopausal (mean age of 65)	800 IU vitamin E and 1000 mg of vitamin C	2.8 years	Higher all-cause mortality in the antioxidant group versus the placebo

SU.VI.MAX: Supplementation en Vitamines et Minéraux Antioxydants; HATS: HDL-Atherosclerosis Treatment Study; WAVE: Women’s Angiographic Vitamin and Estrogen trial.

**Table 3:** Meta-analyses investigating vitamin C and cardiovascular markers.

Meta-Analysis Topic	Number of Studies	Total Participants (*n*)	Trial Sizes (*n*)	Trial Durations	Vitamin C Supplement Dosages	Outcome
Arterial Stiffness [26]	20	1909	8 to 1162	8 h to 2635 days	120 to 4000 mg daily	Arterial stiffness was reduced with vitamin E and combined antioxidant supplement, but not with vitamin C alone
Endothelial Function [27]	46 (17 supplemented with vitamin C alone)	1817 (478 in trials that supplemented with vitamin C alone)	7 to 197	4 to 240 weeks	500 to 2000 mg daily	Significant improvements in endothelial function
Lipid Profile [28]	40	1981	8 to 305	2 to 240 weeks	125 to 4500 mg daily	No significant change in blood lipids
Blood Pressure [29]	29	1407	10 to 120	2 to 26 weeks	60 to 4000 mg daily	Vitamin C supplementation reduced systolic and diastolic blood pressure
